# Behçet’s disease-associated myositis presenting as exercise-induced leg pain

**DOI:** 10.1093/rap/rkae122

**Published:** 2024-10-03

**Authors:** Richard Porter, Pippa Watson, Yee Chiu, Eleftherios Agorogiannis

**Affiliations:** Department of Rheumatology, Manchester Foundation Trust, Manchester, UK; Department of Rheumatology, Manchester Foundation Trust, Manchester, UK; Department of Rheumatology, Manchester Foundation Trust, Manchester, UK; Department of Ophthalmology, Manchester Foundation Trust, Manchester, UK

Key message

- Bechet’s disease can rarely present with myositis and non-specific manifestations, which may mimic medium-/small-vessel vasculitis.

Dear Editor, We describe a case of a 29-year-old gentleman who presented with exercise-induced bilateral lower leg pain and swelling. He was reviewed in emergency care on four occasions, receiving treatment for presumed cellulitis, with US-Doppler studies, excluding thromboembolic disease and plain film excluding fracture. MRI of the lower legs was requested given ongoing symptoms with lack of diagnostic progress. This demonstrated bilateral muscle oedema in the antero-lateral compartments and lateral gastrocnemius heads, prompting rheumatology referral.

Prior to attending a rheumatology clinic, the patient developed bilateral sequential visual loss. This was attributed to right acute macular neuroretinopathy, followed by significant left posterior pole choroidal ischaemia 2 months later. Each presentation was associated with transient low-grade anterior uveitis, vitritis and the presence of a pre-papillary vitreous infiltrate. Initial treatment included a 6-week oral prednisolone taper starting at 60 mg daily; however, there was no secure unifying diagnosis due to atypical features.

On review in Rheumatology, the patient reported progressive leg pain, which was initially noted during cycling but later affecting his walking. There was no reported early morning stiffness or diurnal variation in symptoms. Steroid treatment following the visual loss had concluded a month prior, and his exercise-induced leg pain had progressed throughout this time. He was noted to be of Cypriot heritage, with no family history of autoimmune disease. A focused history revealed several episodes of non-severe, fleeting oral ulceration prior to his leg pain and an acne-like pustular rash over the back, which appeared at the time of his initial visual symptoms. His gait was antalgic with minimal toe-off phase. Investigations demonstrated ESR 52 mm/h and CRP 88 mg/dl, negative autoimmune immunology, negative myositis ENA, creatinine kinase (CK) 237 IU/l and positive HLA-B51. Given these features, a provisional diagnosis of Behçet’s Disease (BD) was made, noting a point score of 5 (ocular lesions 2, oral aphthosis 2, skin lesions 1) using the International Criteria for Behçet’s Disease (ICBD) [1].

Nerve conduction studies displayed non-specific S1 neurogenic changes and small tibial motor amplitudes. MRI lumbar spine was performed to consider and excluded radiculopathy. Repeat CK remained within normal limits and MRI of the orbits, upper arms and thighs revealed no other areas of myositis.

A second episode of visual loss prompted admission under ophthalmology and treatment with intravenous methylprednisolone and azathioprine. Following cross-specialty discussion and liaison with our Regional Behçet’s centre, the patient was commenced on infliximab 5 mg/kg. This treatment, accompanied by a slow oral steroid taper, resulted in stabilization of the disease. Muscle biopsy was sought early; however, due to recurrent admissions was only possible following significant steroid treatment. The right tibialis anterior sample demonstrated a single necrotic fibre but no specific inflammatory component.

This case was notable for the complexity and atypical presenting features with calf pain induced by exercise likely due to atypical myositis. More classical BD symptoms, including oro-genital ulceration, occurred significantly later, and the eye disease was complex and atypical. Following a sustained period of high-dose steroid to allow transition to maintenance treatment, there were no new eye symptoms, resolution of the skin manifestations and significant improvement in the calf symptoms. At this stage, interval MRI of the lower legs was performed, given the lack of complete resolution and a significant improvement in the oedematous changes can be observed (Fig. 1). Yilmaz *et al*. [2] classified typical MRI changes into five discreet categories, but case-report numbers are currently too limited to link to other disease characteristics.

**Figure 1.**
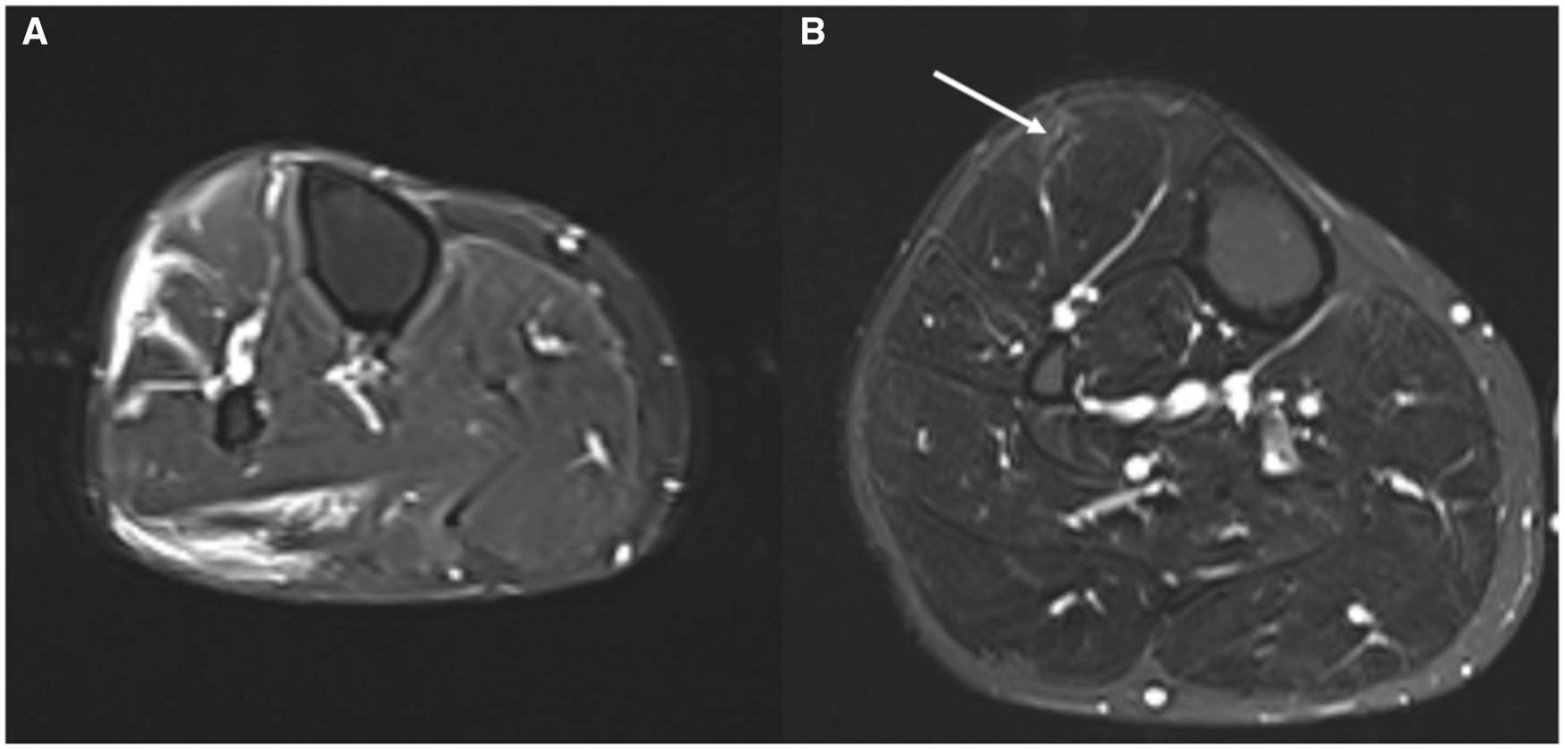
MRI of the lower legs. (**A**) At the time of initial presentation, prior to any treatment, showing diffuse myo-oedematous and myofascial changes. (**B**) Following treatment with steroid, infliximab and azathioprine 6 months later. The white arrow shows a tiny focus of residual oedematous change anterior to the tibialis anterior tendon on the right and the patient continued to report ongoing pain here

Gallay *et al*. [3] retrospectively observed the characteristics of patients with focal myositis, with and without associated BD. In their cohort of patients with BD, myositis was reported in the lower limbs in 60% and accompanied by localized pain, swelling and warmth. CK levels were normal in 80% and only mildly elevated in the remainder. These proportions correlate to findings in Korean [4] and Japanese [5] cohorts, where patients presented with focal myositis prior to a diagnosis of BD. Reported muscle biopsy results from cohort studies are heterogeneous, ranging from minimal non-specific change to lymphocytic infiltrates, eosinophilia, perimysial fibrosis and necrosis. This may well be related to the duration of disease at the time of biopsy. Interestingly, cohort studies largely report treatment efficacy with oral steroid in 1–3 weeks, whereas the present patient’s condition was significantly more refractory to treatment.

One year following initial presentation, there has been sustained improvement in the lower limb muscle symptoms; however, the patient has residual visual deficit owing to the severity of the initial disease flare. This case demonstrates the potential of BD to present with features outside of the ICBD and the need for dissemination of knowledge to allow prompt diagnostic workup.

## Data Availability

No new data were generated or analysed in support of this research.
